# Genome-wide identification of long noncoding RNAs and their competing endogenous RNA networks involved in the odontogenic differentiation of human dental pulp stem cells

**DOI:** 10.1186/s13287-020-01622-w

**Published:** 2020-03-13

**Authors:** Zhao Chen, Kaiying Zhang, Wei Qiu, Yifei Luo, Yuhua Pan, Jianjia Li, Yeqing Yang, Buling Wu, Fuchun Fang

**Affiliations:** 1 Department of Stomatology, Nanfang Hospital, Southern Medical University, Guangzhou, 510515 Guangdong People’s Republic of China; 2 College of Stomatology, Southern Medical University, Guangzhou, 510515 Guangdong People’s Republic of China

**Keywords:** Competing endogenous RNA, Human dental pulp stem cells, Long noncoding RNA, MicroRNA, Odontogenic differentiation

## Abstract

**Background:**

Long noncoding RNAs (lncRNAs) play an important role in the multiple differentiations of mesenchymal stem cells (MSCs). However, few studies have focused on the regulatory mechanism of lncRNAs in the odontogenic differentiation of human dental pulp stem cells (hDPSCs).

**Methods:**

hDPSCs were induced to differentiate into odontoblasts in vitro, and the expression profiles of lncRNAs, microRNAs (miRNAs), and messenger RNAs (mRNAs) in differentiated and undifferentiated cells were obtained by microarray. Bioinformatics analyses including Gene Ontology (GO) analysis, pathway analysis, and binding site prediction were performed for functional annotation of lncRNA. miRNA/odontogenesis-related gene networks and lncRNA-associated ceRNA networks were constructed. Quantitative reverse-transcription polymerase chain reaction (qRT-PCR) was used to verify the expression of selected genes. RNA fluorescence in situ hybridization (FISH), qRT-PCR, and western blot analysis were used to explore the location and function of lncRNA-G043225. Dual-luciferase reporter assay was performed to confirm the binding sites of miR-588 with G043225 and Fibrillin 1 (FBN1).

**Results:**

We identified 132 lncRNAs, 114 miRNAs, and 172 mRNAs were differentially expressed. GO analysis demonstrated that regulation of the neurogenic locus notch homolog (Notch), Wnt, and epidermal growth factor receptor (ERBB) signaling pathways and activation of mitogen-activated protein kinase (MAPK) activity were related to odontogenic differentiation. Pathway analysis indicated that the most significant pathway was the forkhead box O (FoxO) signaling pathway, which is related to odontogenic differentiation. Two odontogenesis-related gene-centered lncRNA-associated ceRNA networks were successfully constructed. The qRT-PCR validation results were consistent with the microarray analysis. G043225 mainly locating in cytoplasm was proved to promote the odontogenic differentiation of hDPSCs via miR-588 and FBN1.

**Conclusion:**

This is the first study revealing lncRNA-associated ceRNA network during odontogenic differentiation of hDPSCs using microarray, and it could provide clues to explore the mechanism of action at the RNA-RNA level as well as novel treatments for dentin regeneration based on stem cells.

## Introduction

Human dental pulp stem cells (hDPSCs) are derived from the ectoderm and originate from neural crest cells, and have the ability to self-renew and undergo multipotential differentiation [1–3]. hDPSCs can differentiate into osteoblasts, chondrocytes, adipocytes, neurons, and odontoblasts [4–8]. Due to their capability to differentiate into odontoblasts, hDPSCs play an important role in dentin repair and regeneration, which provides a new approach for regeneration treatment of dental tissue in clinical applications [9, 10]. However, the specific molecules and mechanisms involved in the odontogenic differentiation process of hDPSCs are still unclear and require further research.

Long noncoding RNA (lncRNA) is defined as a noncoding ribonucleic acid with a length of more than 200 nucleotides and that lacks protein-encoding capacity [11]. Increasing evidence has confirmed that lncRNAs are widely involved in the biological processes including but not limited to DNA methylation, histone modification, and chromatin remodeling and can interact with DNA, RNA, and protein molecules to regulate the expression of target genes via *cis* or *trans* mode [12–14]. Although two lncRNAs have been shown to regulate odontogenesis [15, 16], the roles of most lncRNAs in this process are unknown.

Many studies have shown that the expression of a number of lncRNAs may be related to cell proliferation, apoptosis, migration, and differentiation [17–19]. lncRNAs can regulate gene expression through a variety of mechanisms. For example, lncRNA can directly bind to homologous genomic DNA and RNA, or they can interact with many proteins by forming complex secondary structures and regulate gene expression at the transcriptional, posttranscriptional, and epigenetic levels [20, 21]. In these various mechanisms, lncRNA can act as a sponge to competitively bind to microRNA (miRNA), resulting in changes in the protein level of coding genes at posttranscriptional level [22]. The current study explored the lncRNA, miRNA, and messenger RNA (mRNA) profiles during odontogenic differentiation of hDPSCs using microarray. Gene Ontology (GO) and pathway analysis were carried out to explore the specific functions of lncRNAs. miRNA/target network and lncRNA-associated competing endogenous RNA (ceRNA) network centered on odontogenesis-related genes were constructed using bioinformatics analyses. The quantitative reverse-transcription polymerase chain reaction (qRT-PCR) method was used to validate the microarray results. LncRNA-G043225 was selected for further research according to the lncRNA-associated ceRNA network, and it might be involved in the process of odontogenic differentiation of hDPSCs through its sponge effect. Our research expands the potential lncRNA-associated ceRNA functions during odontogenic differentiation of hDPSCs and enhances the understanding of noncoding RNA (ncRNA) regulatory networks.

## Materials and methods

### hDPSC isolation, culture, and identification

Healthy premolars were extracted from 19 healthy adults (8 males and 11 females, aged from 15 to 25 years old, mean age 19.7) who were undergoing orthodontic treatment at the Department of Stomatology, Nanfang Hospital, Southern Medical University. This study was approved by the Ethics Committee of Nanfang Hospital, Southern Medical University. Written informed consent was obtained from all adult patients. For minor population, statement on informed consent was obtained from their parents. hDPSCs were cultured as previously described [23]. Isolated hDPSCs were cultured in Dulbecco’s modified Eagle’s medium (DMEM) (Gibco, Grand Island, NY, USA) supplemented with 10% fetal bovine serum (FBS) (Gibco, Grand Island, NY, USA) and 100 U/mL penicillin/streptomycin (HyClone, NY, USA), and maintained at 37 °C in a humidified atmosphere with 95% air and 5% CO_2_. A single-cell suspension was obtained by filtering cells through a 70-μm filter (BD Falcon, Franklin Lakes, NJ). Single-cell suspensions were inoculated into six-well plates at a density of 1 × 10^4^ cells/well. Limited dilution techniques were used to obtain colonies of single-cell origin [24].

Cells were characterized by using stem cell surface markers through flow cytometry (Becton Dickinson, Tokyo, Japan). hDPSCs were suspended in phosphate-buffered saline (PBS) containing 2% FBS and incubated with antibodies on ice for 30 min. The following antibodies were used: anti-phycoerythrin (PE), anti-fluorescein iso thiocyanate (FITC), (BD Pharmingen, Franklin Lakes, NJ). Isotype-identical antibodies were used as controls. All procedures were carried out in darkness at 4 °C. The expression profiles were determined by flow cytometer.

### Odontogenic induction

hDPSCs in the differentiated group were cultured with an odontogenic differentiation medium containing 50 mg/mL ascorbic acid, 100 nmol/L dexamethasone, and 10 mmol/L β-glycerophosphate (Sigma, St Louis, MO, USA) for 14 days in 6-well plates. hDPSCs in the undifferentiated group were cultured in 10% FBS in DMEM with no supplements. Then, the cells were rinsed 3 times with PBS, fixed in 4% formaldehyde for 30 min, and stained with 2% Alizarin Red (Alizarin Red S A5533, Sigma-Aldrich) at room temperature for 10 min. After washing several times with deionized water, calcium nodules were observed with a microscope (Crystal violet, Amresco, Solon, OH). For ALP staining, cells were cultured in odontogenic differentiation medium for 7 days and then stained. ALP staining was performed using the NBT/BCIP Staining Kit (Beyotime Biotech, Shanghai, China) following the protocol.

### Preparation of RNA samples

According to the manufacturer’s protocol, total RNA was isolated from cultured cells by TRIzol reagent (Thermo Fisher Scientific, Waltham, MA). The quantity and quality of RNA were determined by using ND-1000 spectrophotometer (NanoDrop Technology Company, Wilmington, DE) and Agilent 2100 Biological Analyzer (Agilent Technologies, Santa Clara, CA).

### lncRNA/mRNA and miRNA microarray

Arraystar Human LncRNA Expression Array V4.0 (KangChen Bio-tech, Shanghai, China) detected 40,173 lncRNAs and 20,730 protein encoded transcripts. Among them, lncRNAs were carefully selected from authoritative public transcriptome databases (RefSeq, UCSC Known Genes, Gencode, etc.) and high impact factor papers. Detailed microarray data about lncRNA and mRNA has been deposited at Gene Expression Omnibus (http://www.ncbi.nlm.nih.gov/geo; GSE138179).

The Whole Human microRNA (miRNA) microarray (KangChen Bio-tech, Shanghai, China) was used to perform expression profiling of all known miRNAs in the human transcriptome. The sequence was compiled from an extensive literature and database survey and then verified and optimized by comparison with the assembled human transcriptome. Detailed microarray data about miRNA has been deposited at Gene Expression Omnibus (http://www.ncbi.nlm.nih.gov/geo; GSE138180).

### Construction of miRNA/targets and ceRNA networks and prediction of binding sites

A regulatory network of differentially expressed miRNAs and odontogenesis-related targets was constructed using the TargetScan and miRanda databases.

**Fig. 1:**
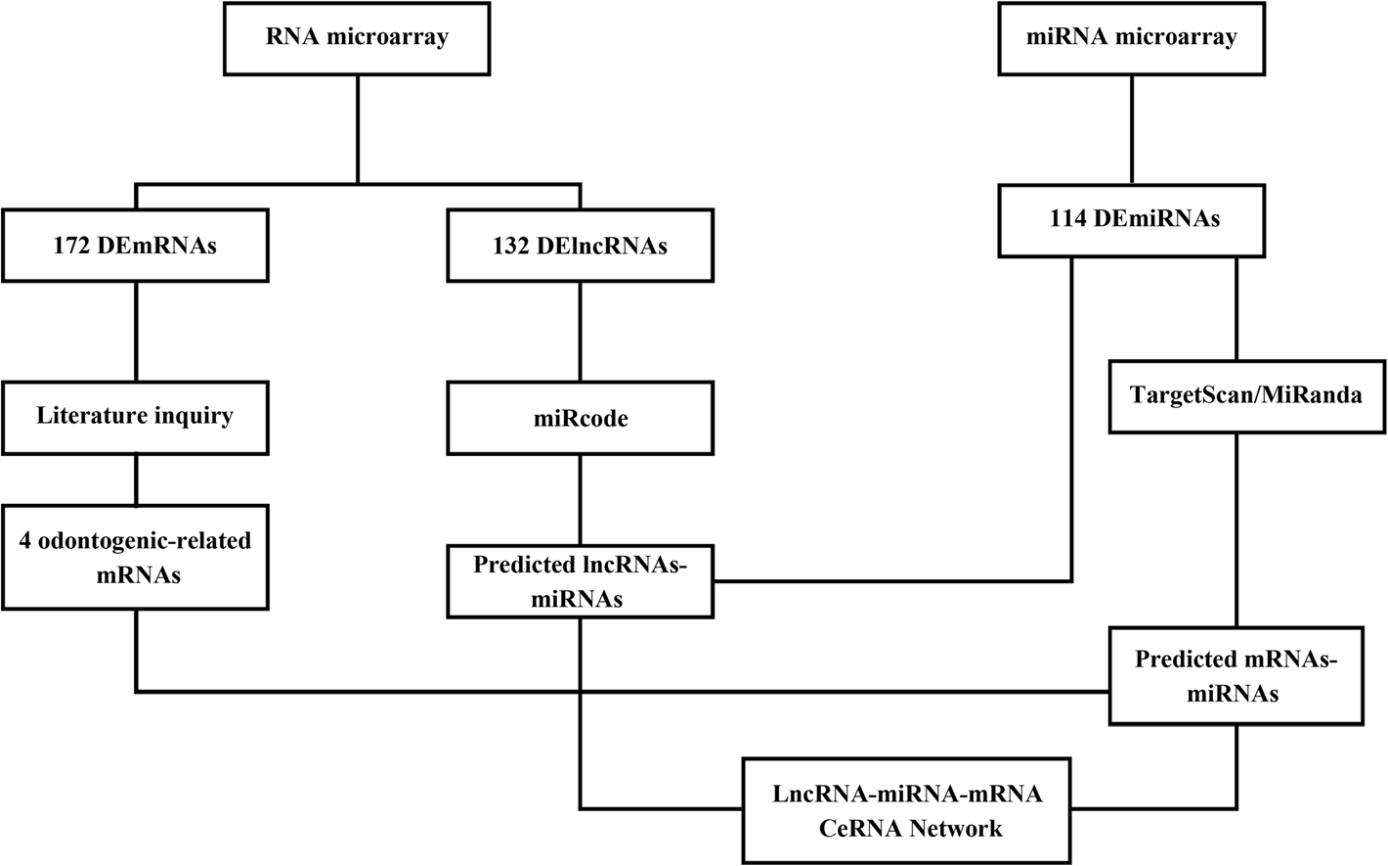
Flow chart of ceRNA regulatory network construction. First, Arraystar Human LncRNA Expression Array V4.0 and the Whole Human miRNA microarray were used to detect differentially expressed lncRNAs, miRNAs, and mRNAs. Second, 4 odontogenesis-related mRNAs were screened by literature. Third, target mRNAs of miRNAs were predicted using TargetScan and miRanda, and target lncRNAs of differentially expressed miRNAs were predicted using miRcode. Then, differentially expressed lncRNAs and mRNAs were merged with the target lncRNAs and mRNAs of differentially expressed miRNAs, respectively. The co-expression lncRNAs and mRNAs were selected (fold change > 2 and *P* < 0.05). Finally, the differentially expressed miRNAs, co-expression lncRNAs, and mRNAs were mapped into the interactions. DERNAs, differentially expressed RNAs; ceRNA, competitive endogenous RNA; lncRNA, long noncoding RNA; miRNA, microRNA; mRNA, messenger RNA

In the ceRNA mechanism, noncoding RNA transcripts can interfere with each other by competing for binding with mRNAs, and RNA reaction elements are the basis of this interaction [25]. These RNA transcripts are called ceRNAs [26]. To find potential targets of miRNAs, target prediction based on TargetScan and miRanda was carried out [27–30]. By merging common target miRNAs, we built ceRNA networks (Fig. 1). The prediction of binding sites between mRNAs and miRNAs, and miRNAs with lncRNAs was carried out by retrieving the TargetScan and miRanda data and was performed by using a homemade software (KangChen Bio-tech, Shanghai, China).

### Functional enrichment analysis

The GO database provides a controlled vocabulary to describe genes and gene product attributes in organisms (http://www.geneontology.org). Ontology covers three fields: biological processes (BP), cellular components (CC), and molecular functions (MF). (*P* value < 0.05 was used). The pathway analysis was based on the Kyoto Encyclopedia of Genes and Genomes (KEGG) pathway database. The *P* value indicates the importance of the path related to the condition (the recommended critical *P* value was 0.05).

### Lentivirus construction and infection

The G043225-specific oligoribonucleotide shRNA-G043225 and control oligoribonucleotide shRNA-ctrl were designed and synthesized by GeneChem Technology (Shanghai, China). The hDPSCs were seeded in 12-well plates at a density of 3 × 10^4^ cells/well and cultured for 24 h. They reached approximately 50% confluence at the time of infection. The cells were infected at a multiplicity of infection (MOI) of 50 in the presence of 5 mg/mL polybrene for 10 h at 37 °C and 5% CO_2_. The knockdown efficiency was evaluated using fluorescence microscopy (Olympus, Tokyo, Japan) and qRT-PCR after infection for 3 days.

### RNA fluorescence in situ hybridization

The RNA fluorescence in situ hybridization (FISH) procedure was performed using the RNA FISH Kit (GenePharma, Shanghai, China) following the manufacturer’s protocol. Briefly, hDPSCs were seeded in 48-well plate and rinsed in PBS, then fixed in 4% paraformaldehyde solution for 15 min at room temperature. Next, cells were incubated with 0.1% Triton X-100 for 15 min. Fluorescence-conjugated G043225 probes were used to perform hybridization in the dark overnight, washed with 0.1% Tween 20 for 5 min and formamide: 2× saline sodium citrate (SSC; 50:50) for 5 min at 42 °C. Cell nuclei were stained with 4′,6-diamidino-2-phenylindole (DAPI) solution. Fluorescence images were obtained by fluorescence microscopy (Olympus, Tokyo, Japan), and representative pictures were shown.

### Dual-luciferase reporter assay

TargetScan online bioinformatics software (http://www.targetscan.org) was used to identify the underlying binding sites of miR-588, G043225, and FBN1. The miR-588 target binding sequences estimated in wild-type and mutant G043225 and FBN1 were synthesized and cloned downstream of luciferase gene in pmirGLO luciferase vector (GeneChem, Shanghai, China). Plasmids were used as standard controls, and luciferase plasmids (GeneChem, Shanghai, China) were transfected into 293T cells using Lipofectamine 2000 (Thermo Fisher Scientific, Waltham, MA, USA). Firefly and renal luciferase activities were continuously measured by dual-luciferase assay after transfection of 48 h.

### qRT-PCR

Total RNA was isolated from cells using TRIzol (Thermo Fisher Scientific, Waltham, MA, USA), and RNA was reverse-transcribed into cDNA by the PrimeScript RT reagent kit (EZBioscience, Roseville, USA). SYBR Green premix Ex Taq (Takara Bio, Tokyo, Japan) was used for subsequent qRT-PCR amplification on an LC480 system. Glyceraldehyde-3-phosphate dehydrogenase (GAPDH) and U6 were used as internal controls. The sequences of gene-specific primers are listed in Table 1.

**Table 1:** Primer sequences for quantitative reverse-transcription polymerase chain reaction

Gene	Sequence 5′ → 3′
RHO	Forward: TCCAGTTTCCCTTGCCAGAC
	Reverse: TGGAGAAGGGGGAGCAGTTA
FBN1	Forward: GCGGAAATCAGTGTATTGTCCC
	Reverse: CAGTGTTGTATGGATCTGGAGC
NKD2	Forward: AGCGCAGATGACGGAGAGA
	Reverse: CGAGACATCGCACTGGAGT
DDIT3	Forward: GGAAACAGAGTGGTCATTCCC
	Reverse: CTGCTTGAGCCGTTCATTCTC
RP11-475I24.3	Forward: AGTGGACGTAGTTATTAAATGCTG
	Reverse: CTGTGAGGACATAGGTGGTGGAG
RP11-17A4.1	Forward: ACGTACACGTTATGCCACCACTG
	Reverse: CACGGAATCCATGCCTGAATCTA
G043225	Forward: GAGCCCGAATGTGACTAAA
	Reverse: GTGGTGGTTGCAGAAGGT
G055009	Forward: TCCTGATGGAGGTGAGCG
	Reverse: GCCTGGGTCCTGGAGAAA
G045900	Forward: GGAGTGGTCTGCTGGAGTTTGC
	Reverse: GGTCCCTGGCCCTCATGTATCC
G019359	Forward: AGGGCACCCAGGACTTCAGATG
	Reverse: TGTGTGGTGGGGTGGTTACTGG
G018548	Forward: TCCGCCTCCTGGGTTCAAGTG
	Reverse: TGGCTGGGTGTGGTGGCTTAC
G012997	Forward: GGGAGGGTCTGGGACTCAAGTG
	Reverse: CCCGCACTGGCACTCACATAC
G003293	Forward: CTCCAGCTCACGTTCCCTA
	Reverse: TCGATGTCGGCTCTTCCT
G000862	Forward: GCTGTGCTCCGCTGAAGACTC
	Reverse: TTGCCTGCTGCCGTGTAAGATG
miR-32-5p	Forward: CCTTGGCCACAATGGGTTAGAAC
miR-588	Forward: CCGCTATTGCACATTACTAAGTTGCA
miR-4717-3p	Forward: TATATAACACATGGGTGGCTGTGGCCT
GAPDH	Forward: CTGGGCTACACTGAGCACC
	Reverse: AAGTGGTCGTTGAGGGCAATG
U6	Forward: CTCGCTTCGGCAGCACA
	Reverse: AACGCTTCACGAATTTGCGT

*RHO* rhodopsin, *FBN1* Fibrillin 1, *NKD2* NKD inhibitor of WNT signaling pathway 2, *DDIT3* DNA damage inducible transcript 3, *GAPDH* glyceraldehyde-3-phosphate dehydrogenase

### Western blotting

Cells were lysed in RIPA buffer (Beyotime, Nanjing, China) supplement with protease inhibitors. Protein samples were separated by sodium dodecyl sulfate polyacrylamide gel electrophoresis (SDS-PAGE) in 15% gel and transferred to polyvinylidene difluoride (Amersham, Little Chalfont, UK) at 200 mA for 2–3 h. The membrane was blocked with 5% skim milk for 1 h and incubated with primary antibody overnight at 4 °C. After washed with Tris-buffer saline containing 0.05% Tween 20 (TBS-T) three times, samples were incubated with the secondary horseradish-peroxidase-conjugated antibody (Proteintech, China). Immunoreactive proteins were visualized by using ECL Kit (Beyotime Biotech, Shanghai, China). The gray value of protein bands was calculated by image J software.

### Statistical analysis

Each data point was expressed as the means ± standard deviation (SD), and the assay was repeated at least three times. Statistical analysis was performed by *t* test and one-way ANOVA using SPSS 17.0 for Windows (SPSS, Chicago, IL, USA). Statistical significance was defined as *P* < 0.05.

## Results

### Characteristics of hDPSCs

**Fig. 2:**
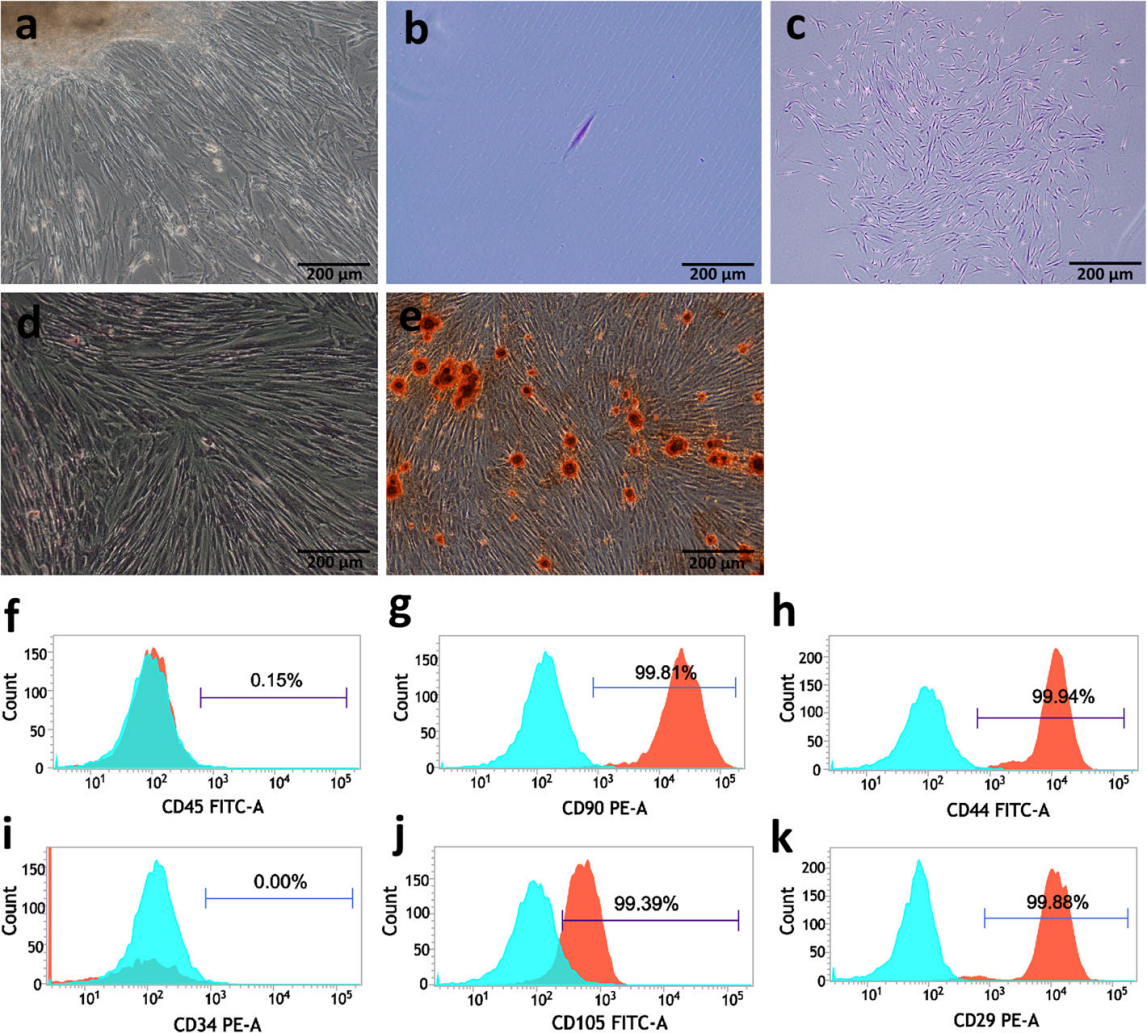
Culture and identification of hDPSCs. **a** Primary cultured hDPSCs (× 100). **b** A single cell was obtained (× 100). **c** Single cell-derived colonies were obtained after culture for 14 days (× 40). Alizarin Red staining was performed to detect mineral nodes in the control group (**d**) and experimental group (**e**) (× 100). **f–k** Flow cytometric analysis of the surface markers of hDPSCs. Cells were incubated with fluorescently conjugated antibodies against CD29, CD90, CD44, CD105, CD34, and CD45. Isotype-identical antibodies served as controls. Analysis of molecular surface antigen markers in hDPSCs by flow cytometry indicated that the cells were positive for CD29, CD44, CD90, and CD105 and negative for CD34 and CD45

After approximately 14 days of culture, the cells emerged from the tissue mass adhering to the culture dish and showed obvious fibroblast-like morphology (Fig. 2a). The hDPSCs used in this study were obtained using limited dilution technique (Fig. 2b, c). After 14 days of odontogenic induction, mineralized nodules at the differentiated and undifferentiated groups could be observed by Alizarin Red staining (Fig. 2d, e). Flow cytometry was used to analyze cell surface markers. The cells were found to be positive for CD29, CD44, CD90, and CD105 and negative for CD34 and CD45 (Fig. 2f–k).

### lncRNA, miRNA, and mRNA profile

**Fig. 3:**
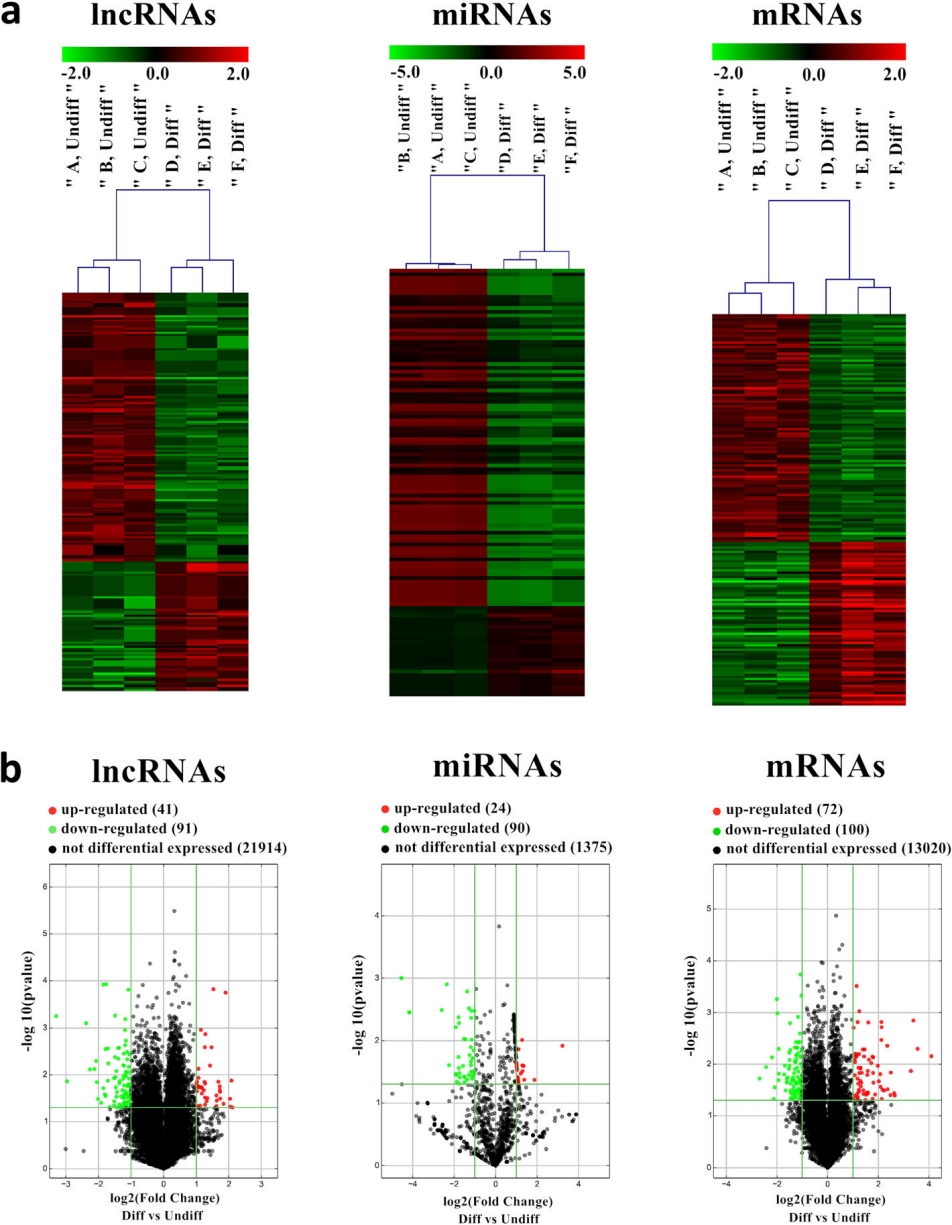
Microarray profiles of lncRNAs, miRNAs, and mRNAs in three groups of hDPSCs. **a** Heatmap of differentially expressed RNAs. Screening criteria were as follows: fold change > 2, *P* < 0.05 for lncRNAs, miRNAs, and mRNAs. The expression value is described by color scale. The intensity increased from green to red. Each column represents one sample, and each row represents one transcript. **b** Volcano map of differentially expressed RNAs. Volcano map reflected the number, significance, and reliability of differentially expressed lncRNAs, miRNAs, and mRNAs. The abscissa is log2 (fold change value), and the ordinate is -log10 (*P* value). Green dots are downregulated genes, red dots are upregulated genes, and gray dots are genes with no significant difference. diff, differentiated; lncRNA, long noncoding RNA; miRNA, microRNA; mRNA, messenger RNA; undiff, undifferentiated

Hierarchical clustering and volcano plot showed that the expression levels of lncRNAs, miRNAs, and mRNAs in the differentiated group differed significantly from those in the undifferentiated group according to fold change (greater than 2) and *P* value (less than 0.05). Among these, 41 lncRNAs were upregulated and 91 were downregulated, 24 miRNAs were upregulated and 90 were downregulated, and 72 mRNAs were upregulated and 100 were downregulated (Fig. 3). The details of dysregulated lncRNAs, miRNAs, and mRNAs are shown in Supplementary Tables 1, 2, 3, 4, 5, and 6.

### Molecular function and pathway prediction

**Fig. 4:**
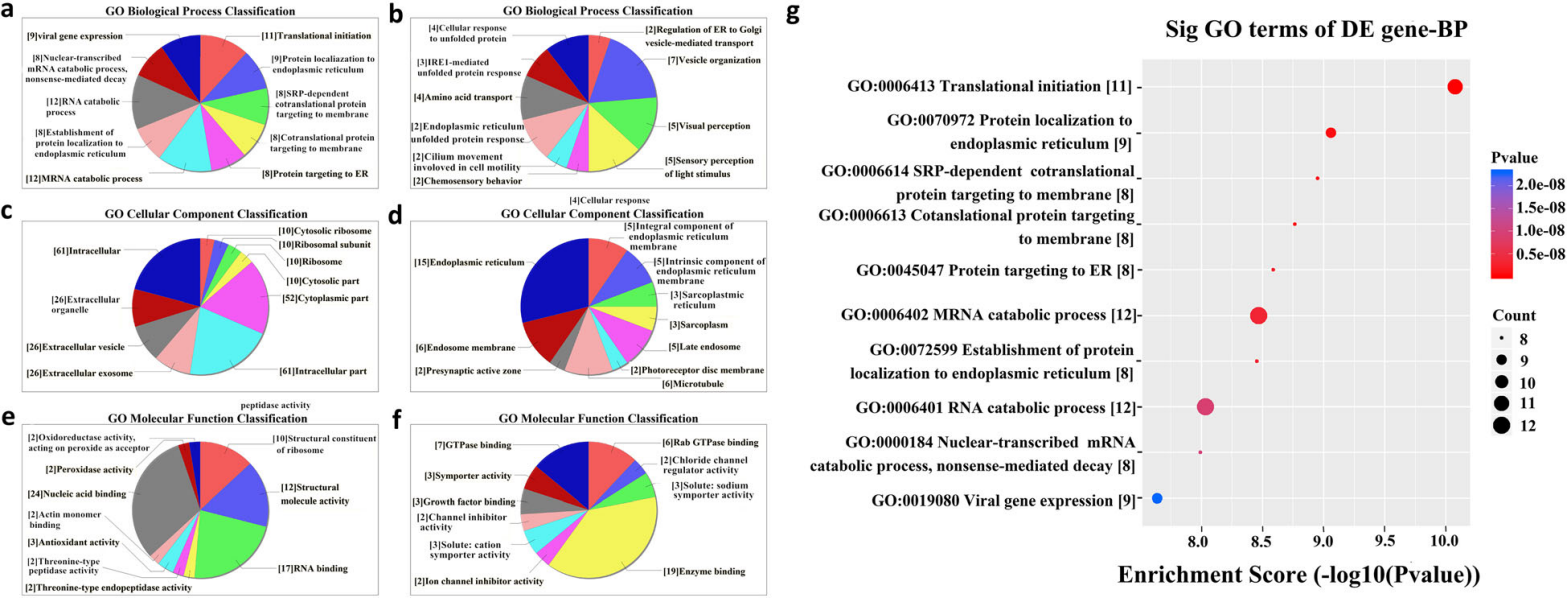
GO annotation analyses of differentially expressed mRNAs. **a** GO biological process classification of upregulated mRNAs. **b** GO biological process classification of downregulated mRNAs. **c** GO cellular component classification of upregulated mRNAs. **d** GO cellular component classification of downregulated mRNAs. **e** GO molecular function classification of upregulated mRNAs. **f** GO molecular function classification of downregulated mRNAs. **g** The enrichment score values of the top ten significantly enriched terms

**Fig. 5:**
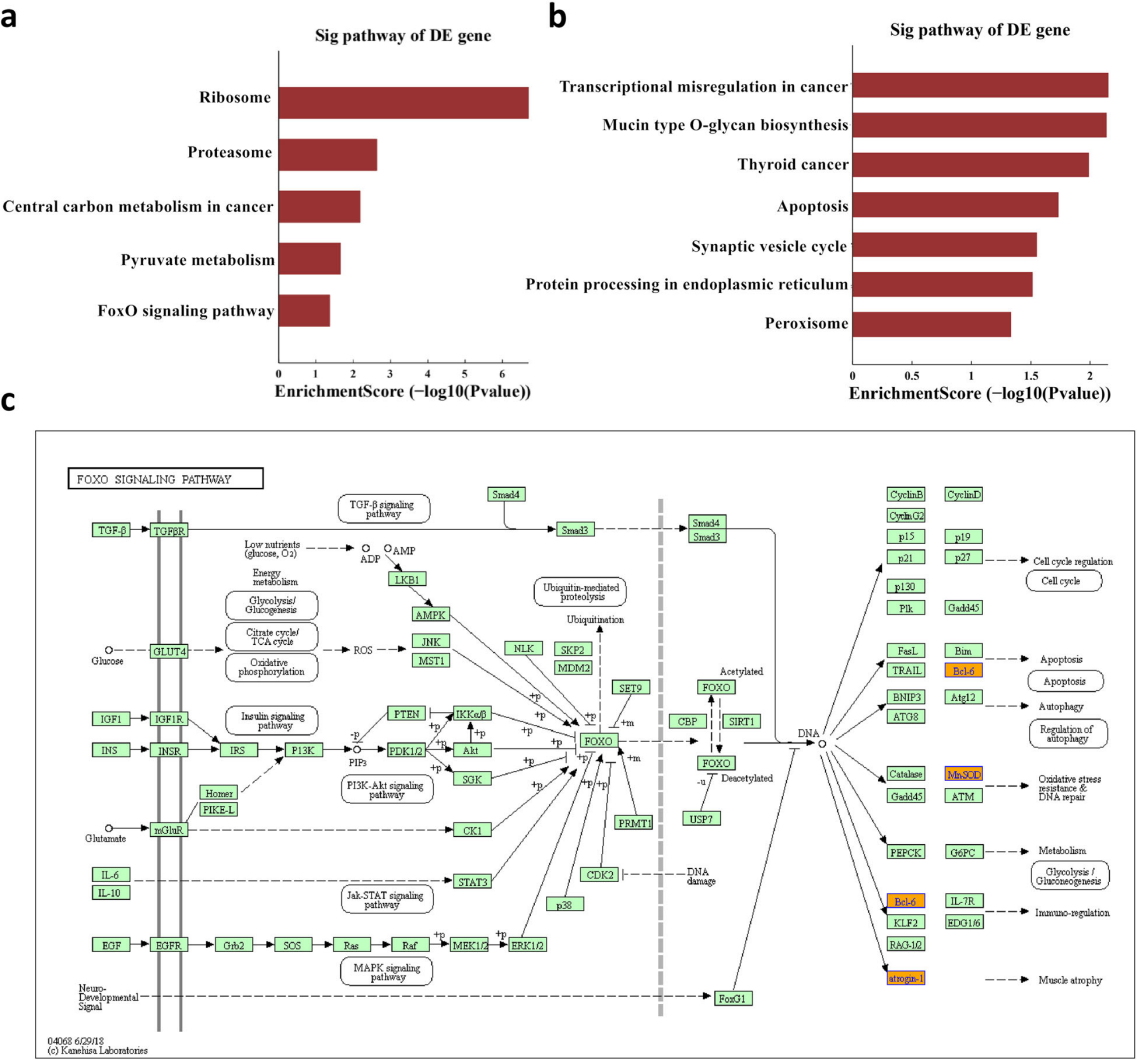
KEGG pathway analyses of differentially expressed mRNAs. **a** KEGG pathway analysis of upregulated mRNAs. **b** KEGG analysis of downregulated mRNAs. **c** FoxO signaling pathway map. Upregulated genes in the microarray results are marked in red. Sig, signal; DE, differentially expressed

The most enriched BP terms related to odontogenic differentiation were regulation of cell differentiation, regulation of Notch signaling pathway, regulation of canonical Wnt signaling pathway, regulation of epidermal growth factor receptor (ERBB) signaling pathway, and activation of mitogen-activated protein kinase (MAPK) activity. The most enriched MF terms were nucleic acid binding, RNA binding, protein binding, etc. The most enriched CC terms were organelle part, intracellular organelle part, endomembrane system, etc. The enrichment score values of the top ten significantly enriched terms are shown in Fig. 4. The most significant pathway was forkhead box O (FoxO) signaling pathway, which is related to odontogenic differentiation (Fig. 5).

### Regulatory network of miRNAs/odontogenesis-related genes

**Fig. 6:**
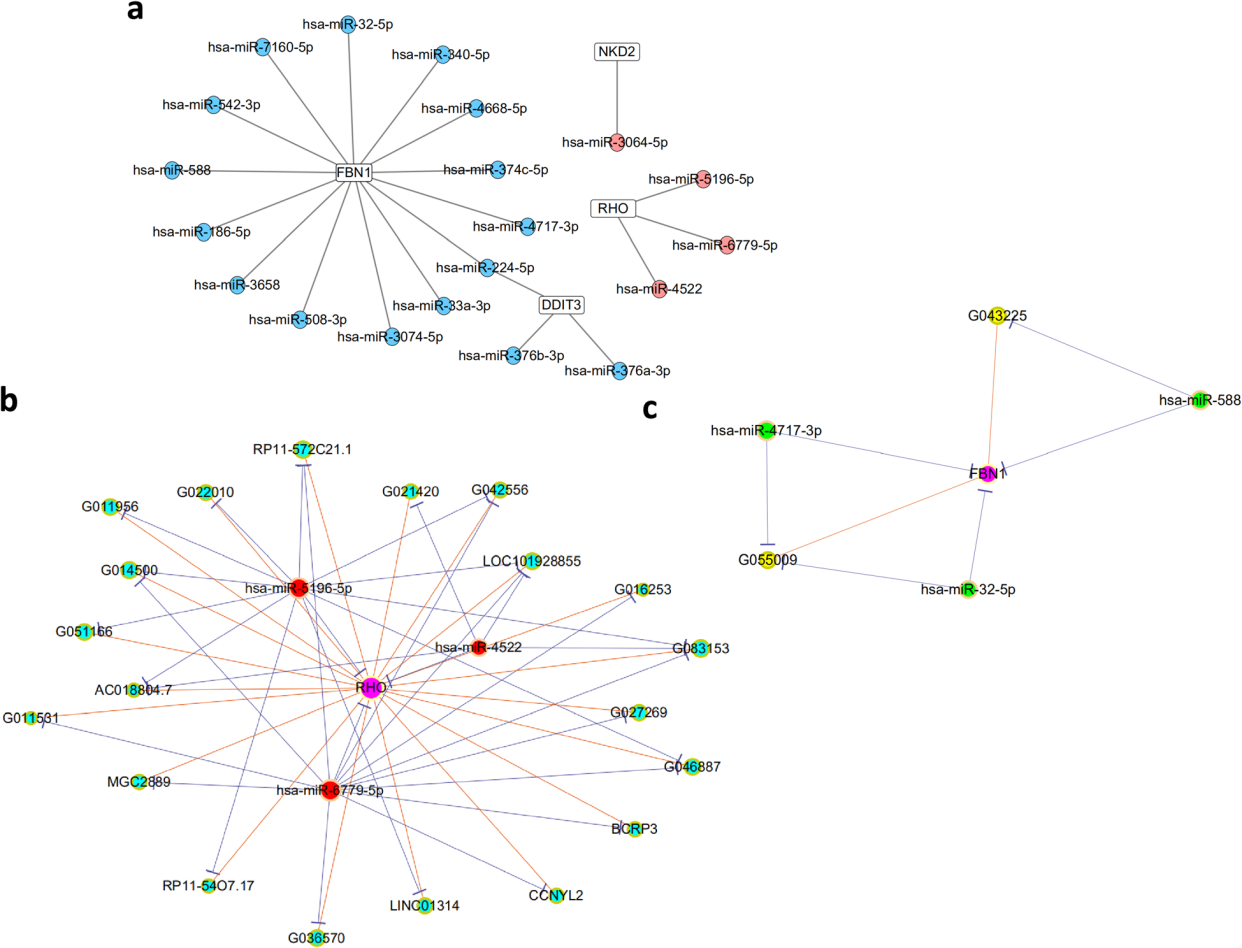
The regulatory networks and their structural characteristics. **a** Regulatory network of miRNAs and odontogenic-related mRNAs. The solid circles colored pale red and light blue represent upregulated and downregulated miRNAs, respectively. The solid squares colored in white represent odontogenic-related mRNAs. **b** View of the ceRNA network for RHO. This network consists of 24 edges among 20 downregulated lncRNA (cyan dots), 3 upregulated miRNAs (red), and 1 downregulated mRNAs (purple). **c** View of the ceRNA network for FBN1. This network consists of 6 edges among 2 upregulated lncRNAs (yellow dots), 3 downregulated miRNAs (green), and 1 upregulated mRNAs (purple)

According to previous reports [31–34], 4 targets related to odontogenesis were identified from the differentially expressed mRNAs. Among the 4 mRNAs, DNA damage inducible transcript 3 (DDIT3) and FBN1 were upregulated, while rhodopsin (RHO) and NKD inhibitor of WNT signaling pathway 2 (NKD2) were downregulated. Twenty miRNAs regulating these 4 targets were predicted using the TargetScan and miRanda algorithm (Fig. 6a).

### lncRNA-miRNA-mRNA network construction and visualization

**Fig. 7:**
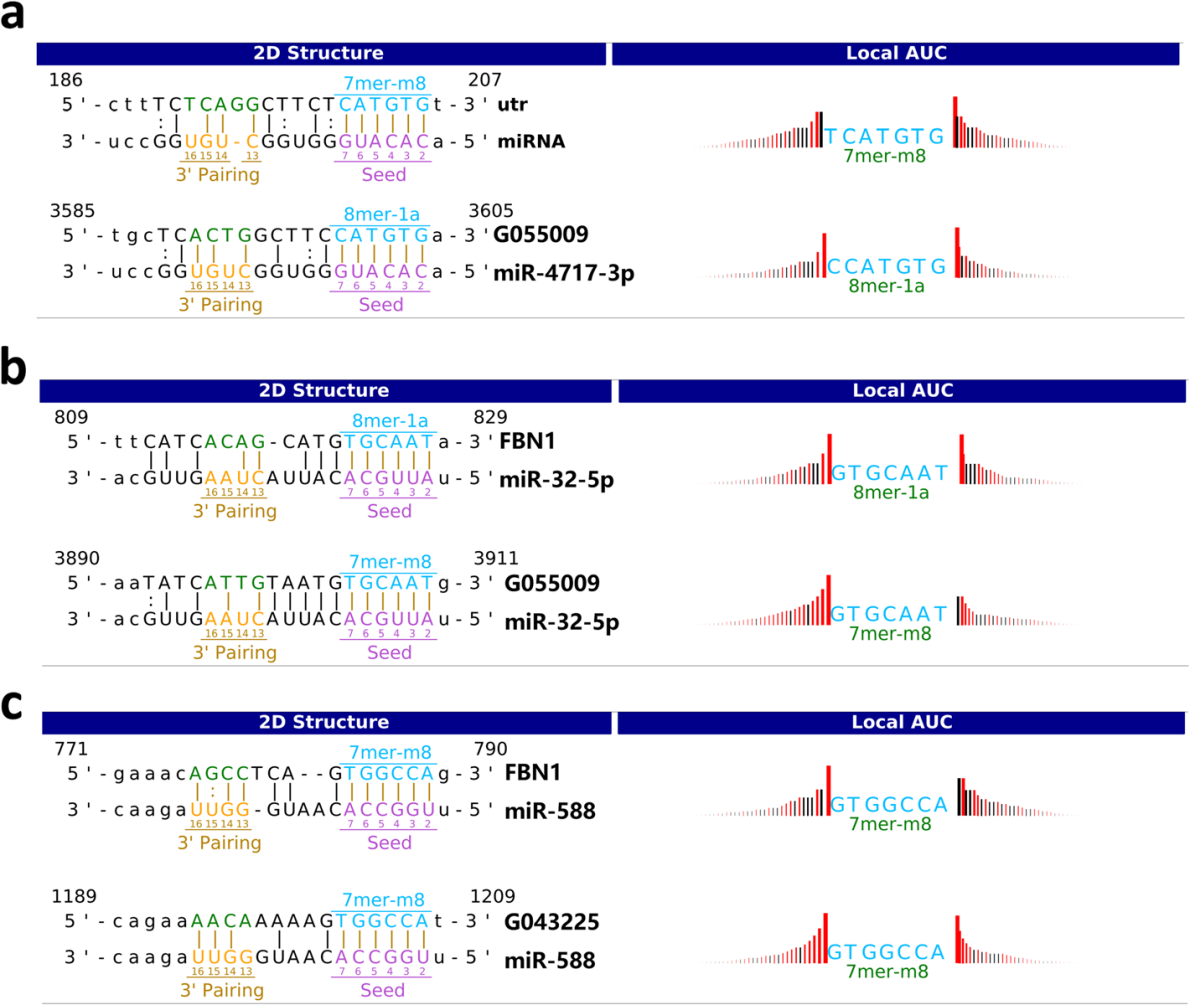
Schematic representation of the putative binding sites of miRNA sequences with mRNA and lncRNA. **a** FBN1 and lncRNA-G055009 binding sequence in the 5′ region of miR-4717-3p. **b** FBN1 and lncRNA-G055009 binding sequence in the 5′ region of miR-32-5p. **c** FBN1 and lncRNA-G043225 binding sequence in the 5′ region of miR-588

Among these 4 odontogenesis-related genes, the upregulated gene FBN1 and the downregulated gene RHO were centered and lncRNA-associated ceRNA networks were successfully constructed (Fig. 6b, c). As shown in Fig. 6c, 2 lncRNAs (G043225 and G055009), 3 miRNAs (miR-4717-3p, miR-32-5p, and miR-588), and FBN1 were included. The binding sites of miR-4717-3p with FBN1 are TCATGTG, while the binding sites of miR-4717-3p with G055009 are CCATGTG. The binding sites of miR-32-5p with FBN1 and miR-32-5p with G055009 share the same sequences GTGCAAT. The binding site sequence of miR-588 with FBN1 and miR-588 with G043225 is GTGGCCA (Fig. 7).

### qRT-PCR validation

**Fig. 8:**
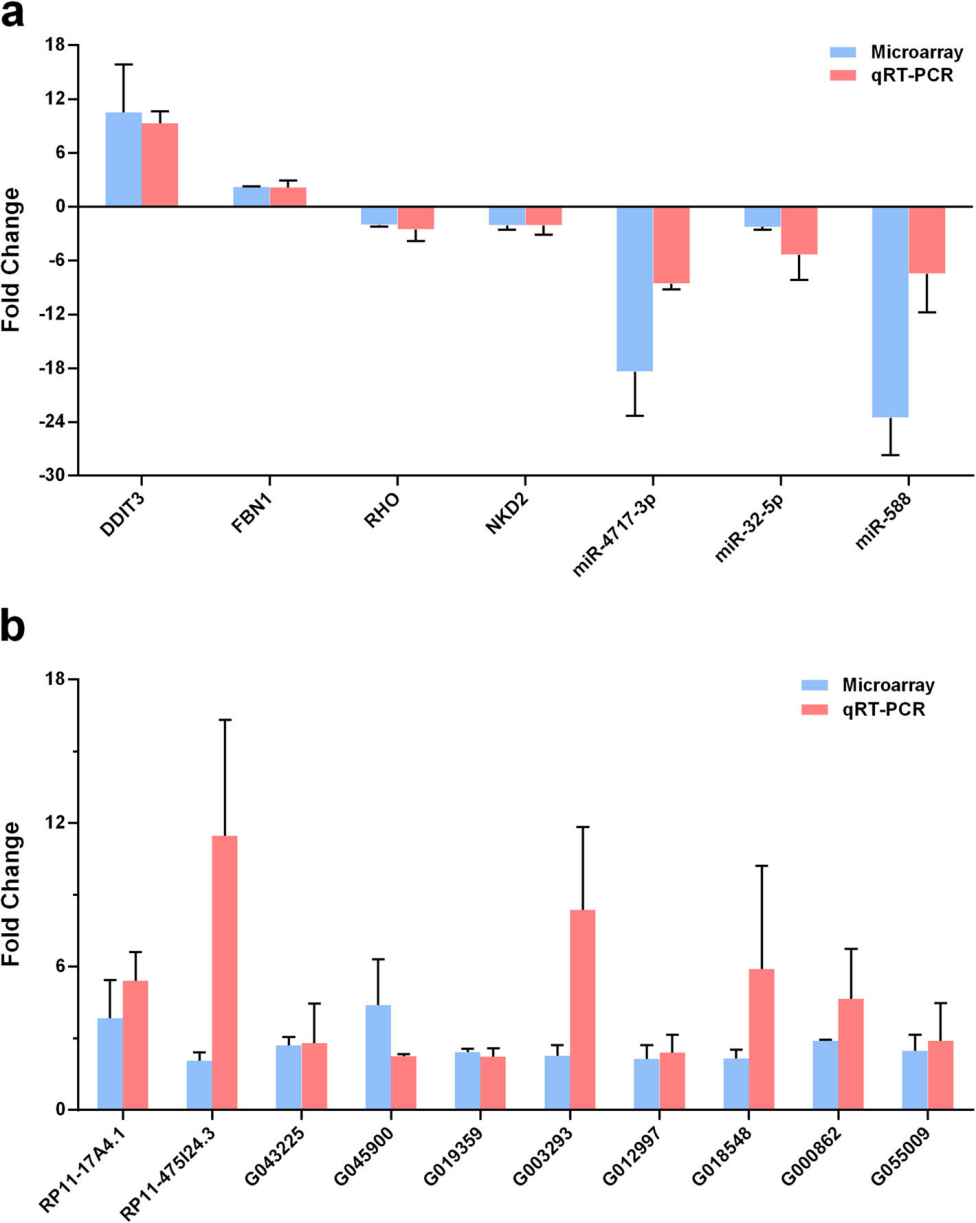
Comparison of microarray and qRT-PCR data for candidate RNAs. **a** Odontogenesis-related mRNAs and miRNAs. **b** Candidate lncRNAs. Heights of the columns in the chart represented mean fold changes in expression for each of the mRNA, miRNAs, and lncRNAs. Four odontogenesis-related mRNAs, three downregulated miRNAs, and ten upregulated lncRNAs were validated by qRT-PCR. Validation results were consistent with the microarray data

Ten differentially expressed lncRNAs, 3 miRNAs, and 4 odontogenesis-related genes were selected and validated using qRT-PCR. The validation results were consistent with the microarray analysis data (Fig. 8).

### The location and function of G043225

**Fig. 9:**
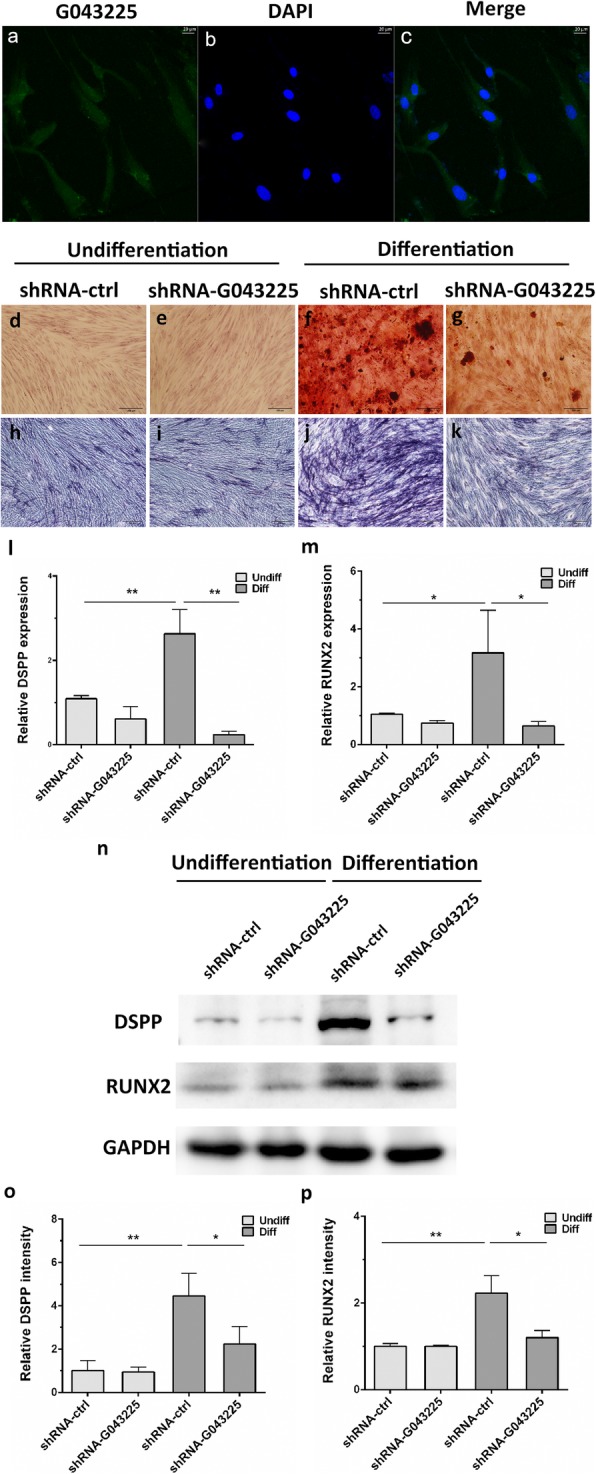
The location and function of G043225. **a**–**c** Expression of G043225 (green) as detected by fluorescence in situ hybridization array. Nuclei (blue) were counterstained with DAPI. **d**–**g** G043225 knockdown inhibited mineralization, indicated by Alizarin Red staining at 14 days after odontogenic induction. **h**–**k** G043225 knockdown inhibited mineralization, indicated by ALP staining at 7 days after odontogenic induction. **l**, **m** The expression level of DSPP and RUNX2 decreased in the shRNA-G043225 group at 14 days after odontogenic differentiation using qRT-PCR. **n**–**p** Western blot analysis shows the expression level of DPSS and RUNX2 decreased in the shRNA-G043225 group after odontogenic differentiation for 14 days. GAPDH was used as an internal control. Data represent means ± SD. *P < 0.05, **P < 0.01 compared with differentiated shRNA-ctrl group

According to the results of qRT-PCR validation and ceRNA network, G043225 was selected for further researches. FISH array revealed that G043225 was mainly distributed in the cytoplasm of hDPSCs (Fig. 9a–c).

ARS staining revealed that knocking down G043225 inhibited the mineralization after 14 days of odontogenic induction (Fig. 9d–g), whereas ALP staining in the shRNA-G043225 group at 7 days was less than the shRNA-ctrl group (Fig. 9h–k). At 14 days of the odontogenic induction, the mRNA expression levels of dentin sialophosphoprotein (DSPP) and RUNX family transcription factor 2 (RUNX2) in the shRNA-G043225 group were significantly lower than the shRNA-ctrl group (Fig. 9l, m). Western blot analyses also confirmed that after 14 days of odontogenic induction, the protein levels of DSPP and RUNX2 were also decreased in the G043225 knockdown group (Fig. 9n–p).

**Fig. 10:**
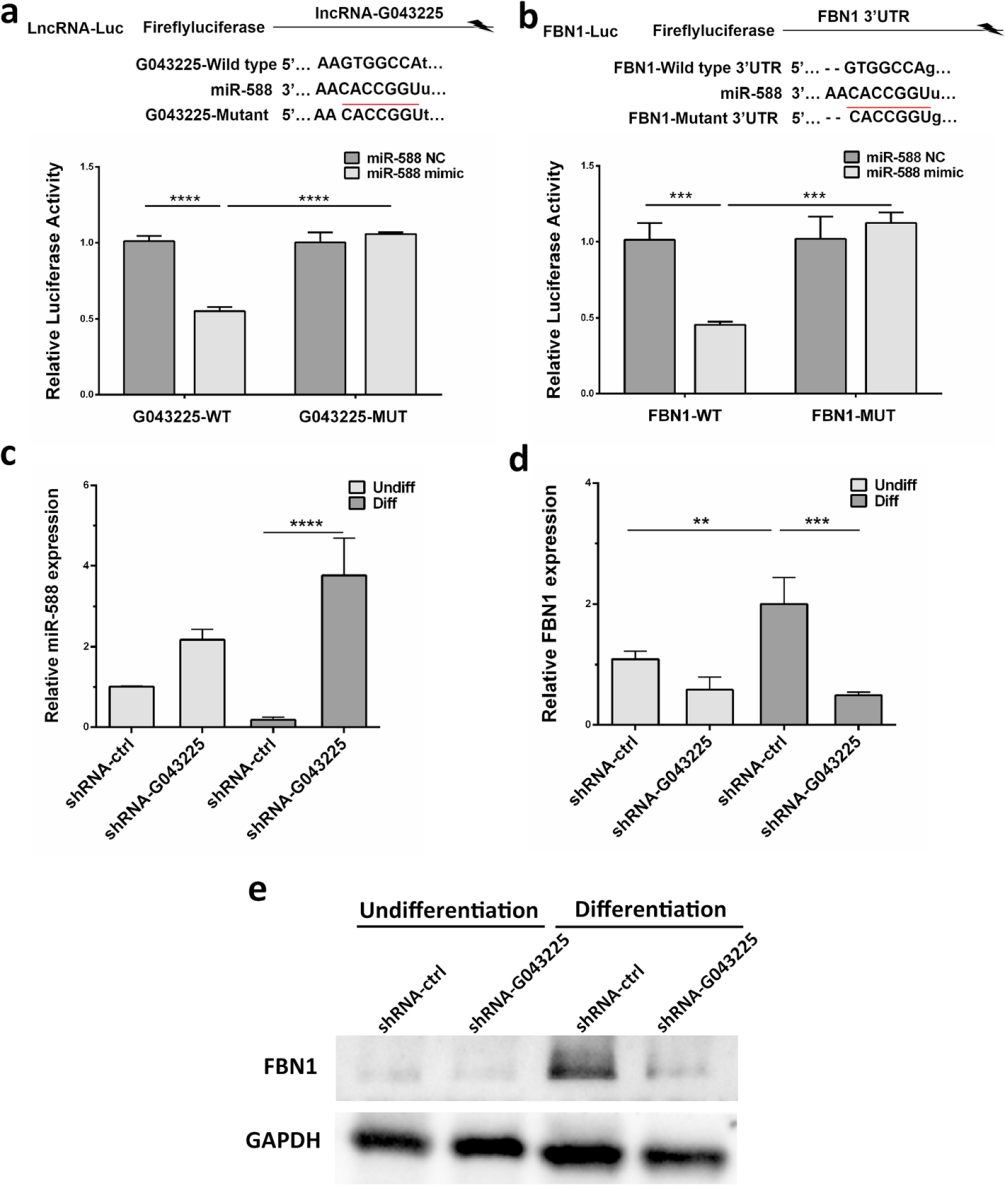
The potential relationship between G043225, miR-588, and FBN1. **a**, **b** Dual-luciferase reporter assay was used to detect the binding sites between G043225 and miR-588, and FBN1 and miR-588. Ectopic expression of miR-588 led to a significant decrease of the reporter luciferase activity with the WT (wild type) but not that of the MUT (mutant) reporter. **c** The increased expression level of miR-588 was determined following interference of G043225 at 14 days after the odontogenic induction by qRT-PCR. **d**, **e** The decreased mRNA and protein expression level of FBN1 was determined in hDPSCs following interference of G043225 at 14 days after the odontogenic induction by qRT-PCR and western blot. GAPDH was used as an internal control. Data represent the mean ± S.D. *P < 0.05, **P < 0.01, ***P < 0.001, ****P < 0.0001 compared with differentiated shRNA-ctrl group

Furthermore, the results of the dual-luciferase reporter showed that miR-588 overexpression significantly decreased the luciferase activity of G043225-WT and FBN1-WT (Fig. 10a, b), indicating that miR-588 could directly bind with both G043225 and FBN1. In addition, the results of qRT-PCR and western blot analysis revealed that the G043225 silencing upregulated miR-588 and downregulated FBN1 at mRNA and protein levels (Fig. 10c–e).

## Discussion

lncRNA-associated ceRNA plays an important role in cell differentiation. Feng et al. [35] reported that linc-ROR promotes osteogenic differentiation of mesenchymal stem cells (MSCs) by functioning as a ceRNA for miR-138 and miR-145. Jia et al. [30] reported LINC00707 sponges miR-370-3p to promote osteogenesis of human bone marrow-derived MSCs through upregulating WNT2B, indicating that ceRNA may play a significant role in the process of cell differentiation, but its role in the process of odontogenic differentiation needs further exploration. To our knowledge, this is the first study to report on lncRNA-associated ceRNA regulation in the odontogenic differentiation of hDPSCs using microarray. lncRNAs often express and function at low abundance, buried in other classes of abundant RNAs. There are serious limitations and many errors in the process of RNA-Seq data analysis, exon detection, and RNA quantification [36, 37]. The microarray annotation and analyses are rich, detailed, and comprehensive, unrivaled by any other profiling platforms, so it is still the preferred platform for lncRNA expression profile detection [38, 39]. Therefore, in this study, we used the microarray to detect lncRNA, miRNA, and mRNA expression. Our findings provide an explanation for the mechanism role of lncRNA in odontogenesis. Further in-depth functional investigations would extend our knowledge of the roles of lncRNA-miRNA-mRNA networks in the odontogenic differentiation of hDPSCs.

When dental pulp is stimulated by infection or trauma, hDPSCs can form new odontoblasts and reparative dentine, which can protect dental pulp from further damage [40]. In the research on ncRNAs and their roles in the odontogenic differentiation of hDPSCs, the most studied are miRNAs. miR-223-3p and miR-21 have been shown to promote odontogenic differentiation, while miR-143-5p, miR-140-5p, and miR-488 inhibit this process [41–46]. In 2016, Chen et al. found that lncRNA differentiation antagonizing non-protein coding RNA (DANCR) inhibited the odontogenic differentiation of human dental pulp cells by inactivating the Wnt/β-catenin signaling pathway [16]. One recent study revealed that lncRNA H19 promoted the odontogenic differentiation of hDPSCs. The author further found that the H19/SAHH axis could epigenetically regulate odontogenic process [15]. To date, few studies have investigated the lncRNA-associated ceRNA networks during odontogenic differentiation of hDPSCs.

The lncRNA-associated ceRNA mechanism has been demonstrated in the multilineage differentiation of MSCs. In this study, we used a genome-wide microarray to detect the differentially expressed lncRNAs, miRNAs, and mRNAs between differentiated and undifferentiated hDPSCs, and focused on the crosstalk between lncRNAs and miRNAs to provide insights into ncRNA coordination for odontogenesis regulation. According to previous reports, four odontogenesis-related genes were identified. Among the four target genes, FBN1 and RHO were used to successfully construct specific ceRNA networks with the corresponding miRNAs and lncRNAs. However, exploration of the ceRNA mechanism of lncRNAs in previous literature indicated that the subcellular localization of lncRNAs is rarely mentioned, even though this localization is the basis of the ceRNA mechanism. For example, if a lncRNA is mainly located in the nucleus, it will not play a major regulatory role through a ceRNA mechanism [47, 48]. In further research concerning the ceRNA mechanism, the subcellular localization of lncRNAs in hDPSCs must be demonstrated.

In addition, miRNA targets and lncRNA-associated ceRNA networks were constructed, and this could provide clues to explore the mechanism of action at the RNA-RNA level. The verification of differentially expressed lncRNAs, miRNAs, and mRNAs by qRT-PCR further improved the credibility of our network. However, we should also take into account that in addition to these four target genes, other significantly differentially expressed target genes might also play an important regulatory role. For example, B cell lymphoma 6 (BCL6) and superoxide dismutase 2 (SOD2) are related to the FoxO signaling pathway according to the KEGG analysis. These two genes have been reported to be involved in osteogenic differentiation [49–51]. Therefore, we assume that these two genes might have potential role in the odontogenic differentiation of hDPSCs, but further exploration is needed.

GO analysis indicated that several important terms relating to odontogenic differentiation were enriched. They included the regulation of cell differentiation, regulation of Notch signaling pathway, regulation of canonical Wnt signaling pathway, and activation of MAPK activity [52–54]. Furthermore, in order to elucidate the potentially relevant signaling pathway, KEGG pathway analysis was used to show the relationships between pathways and genes. This analysis found that FoxO signaling pathway was enriched in odontogenic-differentiated hDPSCs [51]. The results revealed that this pathway might play a crucial role in the odontogenic differentiation of hDPSCs, but the functions need further exploration.

In our research, lncRNA DANCR and H19 expression were not be significantly detected. There are several reasons for this result. First, the differentiated and undifferentiated cells were derived from different people in different studies, which might result in different results. Second, the phenotypic differences can affect the results. Third, different sequencing methods might contribute to the difference. Therefore, large-scale studies are needed to confirm the results.

To further elucidate the ceRNA network, the function and potential mechanism of G043225 were explored. Functional assays showed the promoting role of G043225 in odontogenic differentiation of hDPSCs. The results that G043225 was mainly located in cytoplasm from FISH array further suggested G043225 might play an important role during the odontogenic differentiation of hDPSCs through ceRNA mechanism. Moreover, the target miR-588 was upregulated and its downstream FBN1 was downregulated after interfering G043225. The results of dual-luciferase reporter revealed miR-588 could directly bind with both G043225 and FBN1. The above data demonstrated that G043225-related ceRNA network played an important role in the odontogenic differentiation of hDPSCs. The function of miR-588 and related rescue experiments are warranted for further validation.

## Conclusion

In summary, to the best of our knowledge, this is the first study revealing the lncRNA-associated ceRNA network during odontogenic differentiation of hDPSCs using microarray. Although the specific role and mechanism of the candidate lncRNAs in the odontogenic differentiation of hDPSCs require further investigation, our findings extended the current knowledge on the odontogenic process of hDPSCs and suggested directions for further investigations on the regulation of odontogenic differentiation of hDPSCs as well as treatments for dentin regeneration based on stem cells.

## Supplementary information

**Additional file 1: Table S1.** The expression profiles of upregulated lncRNAs in differentiated and undifferentiated hDPSCs. (XLS 72 kb)

**Additional file 2: Table S2.** The expression profiles of downregulated lncRNAs in differentiated and undifferentiated hDPSCs. (XLS 119 kb)

**Additional file 3: Table S3.** The expression profiles of upregulated miRNAs in differentiated and undifferentiated hDPSCs. (XLS 33 kb)

**Additional file 4: Table S4.** The expression profiles of downregulated miRNAs in differentiated and undifferentiated hDPSCs. (XLS 63 kb)

**Additional file 5: Table S5.** The expression profiles of upregulated mRNAs in differentiated and undifferentiated hDPSCs. (XLS 88 kb)

**Additional file 6: Table S6.** The expression profiles of downregulated mRNAs in differentiated and undifferentiated hDPSCs. (XLS 108 kb)

## Data Availability

The data supporting the research results obtained from the corresponding authors according to reasonable requirements.

## Abbreviations

BCL6: B cell lymphoma 6
BP: Biological processes
CC: Cellular components
ceRNA: Competitive endogenous RNA
DANCR: Differentiation antagonizing non-protein coding RNA
DDIT3: DNA damage inducible transcript 3
DE: Differentially expressed
DERNAs: Differentially expressed RNAs
DMEM: Dulbecco’s modified Eagle’s medium
DSPP: Dentin sialophosphoprotein
ERBB: Epidermal growth factor receptor
FBN1: Fibrillin 1
FBS: Fetal bovine serum
FISH: Fluorescence in situ hybridization
FoxO: Forkhead box O
GAPDH: Glyceraldehyde-3-phosphate dehydrogenase
GO: Gene Ontology
hDPCs: Human dental pulp cells
hDPSCs: Human dental pulp stem cells
KEGG: Kyoto Encyclopedia of Genes and Genomes
lncRNAs: Long noncoding RNAs
MAPK: Mitogen-activated protein kinase
MF: Molecular functions
miRNAs: MicroRNAs
mRNA: Messenger RNA
MSCs: Mesenchymal stem cells
ncRNAs: Noncoding RNAs
NKD2: NKD inhibitor of WNT signaling pathway 2
Notch: Neurogenic locus notch homolog
PBS: Phosphate buffer solution
qRT-PCR: Quantitative reverse-transcription polymerase chain reaction
RHO: Rhodopsin
RUNX2: RUNX family transcription factor 2
SD: Standard deviation
Sig: Signal
SOD2: Superoxide dismutase 2
